# Increased 15-lipoxygenase-1 expression in chondrocytes contributes to the pathogenesis of osteoarthritis

**DOI:** 10.1038/cddis.2017.511

**Published:** 2017-10-12

**Authors:** Kaizhe Chen, Yufei Yan, Changwei Li, Jun Yuan, Fei Wang, Ping Huang, Niandong Qian, Jin Qi, Hanbing Zhou, Qi Zhou, Lianfu Deng, Chuan He, Lei Guo

**Affiliations:** 1 Shanghai Key Laboratory for Bone and Joint Diseases, Shanghai Institute of Orthopaedics and Traumatology, Shanghai Ruijin Hospital, Shanghai Jiao Tong University School of Medicine, Shanghai, China; 2 Department of Orthopaedics, Ruijin Hospital, Shanghai Jiao Tong University School of Medicine, Shanghai, China

## Abstract

15-Lipoxygenase-1 (15-LO-1) is involved in many pathological processes. The purpose of this study was to determine the potential role of 15-LO-1 in osteoarthritis (OA). The levels of 15-LO-1 expression were measured by western blotting and quantitative real-time PCR in articular cartilage from the OA rat models and OA patients. To further investigate the effects of 15-LO-1 on chondrocyte functions, such as extracellular matrix (ECM) secretion, the release of matrix-degrading enzymes, the production of reactive oxygen species (ROS), cell proliferation and apoptosis, we decreased or increased 15-LO-1 expression in chondrocytes by means of transfecting with siRNA targeting 15-LO-1 and plasmid encoding 15-LO-1, respectively. The results showed that 15-LO-1 expression was obviously increased in articular cartilage from OA rats and OA patients. It was also found that many factor-related OA, such as mechanical loading, ROS, SNP and inflammatory factor, significantly promoted 15-LO-1 expression and activity in chondrocytes. Silencing 15-LO-1 was able to markedly alleviate mechanical loading-induced cartilage ECM secretion, cartilage-degrading enzyme secretion and ROS production. Overexpression of 15-LO-1 could inhibit chondrocyte proliferation and induce chondrocyte apoptosis. In addition, reduction of 15-LO-1 *in vivo* significantly alleviated OA. Taken together, these results indicate that 15-LO-1 has an important role in the disease progression of OA. Thus 15-LO-1 may be a good target for developing drugs in the treatment of OA.

Osteoarthritis (OA), the most common chronic joint disease, affects millions of patients worldwide. OA characterized by degenerative alternation in the morphology, composition and mechanical properties of the articular cartilage lead to chronic pain, joint instability, stiffness, joint deformities and radiographic joint space narrowing, resulting in the progressive disability and reduction of patient’s quality of life.^1^ The prevalence of OA increases significantly with age, with radiographic evidence observed in >70% of the population aged >65 years.^2^ Multifactorial etiopathogenesis were associated with the progression of OA, such as genetic predisposition, aging, obesity, and joint malalignment.^3^ Considering the extensive impact and consequences of this disease, at present there is no intervention that has been proven to prevent the development and progression of OA.

Articular cartilage is a unique and a vascular connective tissue that covers diarthrodial joint surfaces, which is mainly composed by tissue fluid, type II collagen (Col2) and proteoglycans. Articular chondrocyte, as the sole cell type in cartilage, is the sensor of articular cartilage homeostasis and has a critical role in maintaining the normal physiological structure and function of articular cartilage.^4^ Chondrocytes are responsible for the synthesis, maintenance and degradation of the cartilage extracellular matrix (ECM), which protects the tissue against tensile stress and physical loading.^5, 6, 7^ Previous studies demonstrated that articular chondrocyte homeostasis could be disrupted by multiple factors, such as abnormal mechanical loading, pro-inflammatory mediators and oxidative stress.^1, 3, 8^ Thus identification of the changes in chondrocytes is critical for understanding the pathogenesis of OA and may lead to the development of new therapeutic targets for the treatment of the disease.

15-Lipoxygenase-1 (15-LO-1) belongs to a lipoxygenase family of enzymes that catalyze the stereospecific addition of molecular oxygen across double bonds in polyunsaturated fatty acids to form a series of biologically active lipid mediators.^9^ 15(*S*)-hydroperoxyeicosatetraenoic acid (15(*S*)-HETE) and 13(*S*) hydroperoxyoctadecadienoic acid (13(*S*)-HODE) are primary metabolites of arachidonic acid and linoleic acid catalyzed by 15-LO-1, respectively. In addition to free fatty acids, more complex substrates are also oxidized by15-LO-1, such as biological membranes, phospholipids, cholesterol esters and plasma lipoproteins.^10^

15-LO-1 is expressed in a variety of tissues and is involved in many pathological processes.^11^ Several studies have documented that 15-LO-1 and its metabolites were associated with inflammatory-related disease, such as arthritis.^12^ Furthermore, Chabane *et al.*^13^ found that 15-HETE, the 15-LO-1 metabolite, suppressed interleukin-1 beta (IL-1*β*)-induced matrix metalloproteinase (MMP)-1 and MMP-13 expression and type II collagen cleavage, suggesting that 15-LO-1 may have a role in the pathogenesis of OA. In addition, 15-LO-1 was confirmed to involve in the regulation of cell function.^14, 15^ However, the roles and the exact mechanisms of 15-LO-1-mediated chondrocyte function in the pathogenesis OA need to be further investigated.

In the present study, we found that the levels of 15-LO-1 expression were increased in the articular knee cartilage from OA patients and OA rat model. 15-LO-1 expression and activity were also significantly upregulated by stimulatory factor-related OA in chondrocyte. Further studies showed that 15-LO-1 was involved in the regulation of chondrocytes functions, including ECM secretion, cartilage-degrading enzyme secretion, the reactive oxygen species (ROS) production, chondrocytes proliferation and apoptosis. In addition, reduction of 15-LO-1 *in vivo* could markedly alleviate OA. Taken together, these data suggest that 15-LO-1 may be a crucial factor for the pathogenesis of OA.

## Results

### The expression of 15-LO-1 is increased in knee articular cartilage from OA patients

**Figure 1:**
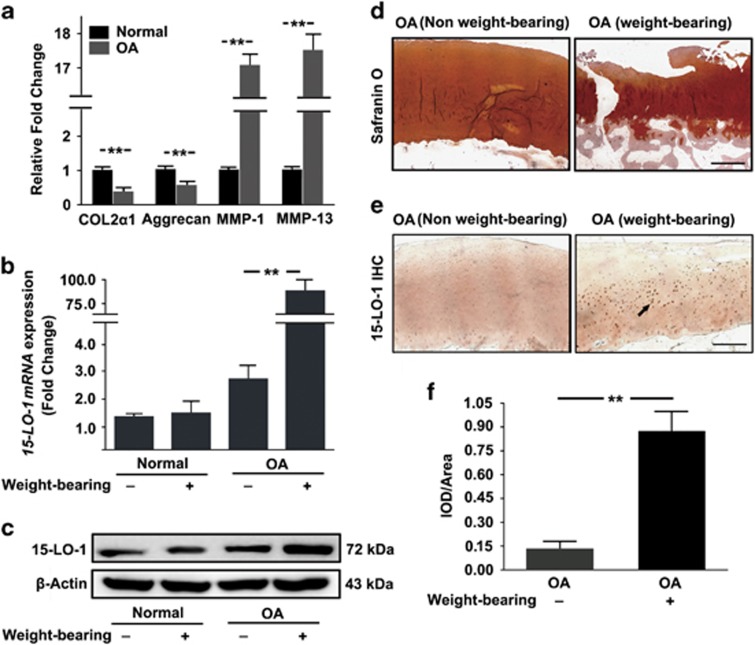
The expression of 15-LO-1 is increased in knee articular cartilage from OA patients. (**a**) QRT-PCR analysis of *COL2α1*, *Aggrecan, MMP-1* and *MMP-13 mRNA* expression in articular knee cartilage obtained from OA patients and normal persons. *n*=4, **P<0.01. (**b**) QRT-PCR analysis of *15-LO-1 mRNA* expression in the weight-bearing area/non-weight-bearing area of articular cartilage from normal persons and OA patients. *n*=4, **P<0.01. (**c**) Western blotting analysis of *15-LO-1* expression in the weight-bearing area/non-weight-bearing area of articular cartilage from normal persons and OA patients. (**d**) The weight-bearing area/non-weight-bearing area of articular cartilage from OA patients were stained with Safranin-O. Scale bar=200 *μ*m. (**e**) Expression and localization (black arrow) of 15-LO-1 were observed by immunohistochemistry staining in the weight-bearing area/non-weight-bearing area of articular knee cartilage derived from OA patients. Scale bar=200 *μ*m. *n*=4. (**f**) Quantitative analysis of positive staining. The intensity of the immunostaining of 15-LO-1 was markedly increased in weight-bearing area. *n*=4, **P<0.01

To explore whether 15-LO-1 was involved in the pathogenesis of OA, we first observed the expression of 15-LO-1 in the knee articular cartilage from OA patients. Quantitative real-time PCR (qRT-PCR) analysis showed that the *mRNA* expression of COL2*α*1 and aggrecan were significantly decreased in OA patients compared with normal persons, whereas MMP-1 and MMP-13 *mRNA* expression were upregulated in OA patients, suggesting that OA patients’ samples were reliable (Figure 1a). Next we examined the expression of 15-LO-1 using qRT-PCR and western blotting in normal persons and OA patients. We found that the expression of 15-LO-1 was obviously upregulated in knee articular cartilage from OA patients, whereas more obvious increasing was observed in weight-bearing area compared with non-weight-bearing area (Figures 1b and c). Safranin O staining showed that the weight-bearing area of articular cartilage from OA patients suffered more severe destruction and loss of integrity compared with non-weight-bearing area (Figure 1d). Subsequently, immunohistochemistry staining was applied to investigate the localization and expression of 15-LO-1 in weight-bearing area of knee articular cartilage from OA patients. The intensity of the immunostaining of 15-LO-1 was markedly increased in weight-bearing area (Figures 1e and f). Taken together, these results indicated that the expression of 15-LO-1 was upregulated in OA, especially in weight-bearing area.

### The expression of 15-LO-1 is increased in knee articular cartilage from OA rat

**Figure 2:**
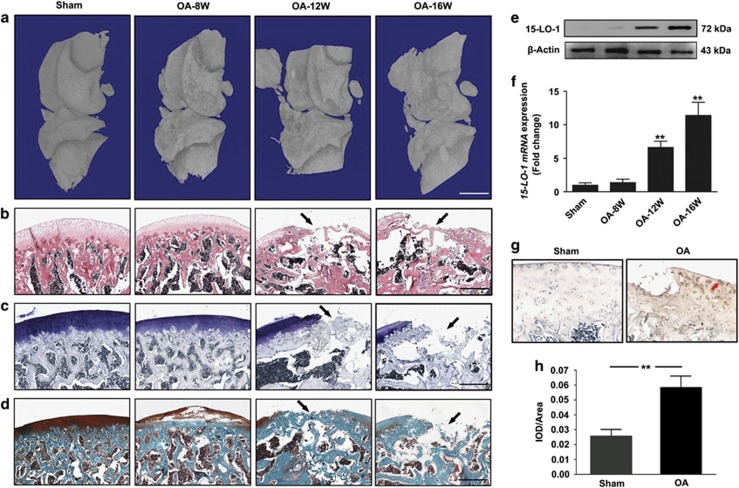
The expression of 15-LO-1 is increased in knee articular cartilage from OA rat. (**a**) Representative micro-CT views of the intact knee of OA rat model at 8-, 12- and 16-week old. Scale bar=1 mm. (**b**–**d**) The articular cartilage at the distal femur metaphysis was stained with hematoxylin (**b**), Toluidine blue (**c**) and Fast Green and Safranin-O (**d**). Scale bar=200 *μ*m. (**e**) Western blotting analysis of 15-LO-1 expression in knee articular cartilage from OA rat at different time courses after surgery. *n*=4. (**f**) QRT-PCR analysis of *15-LO-1* expression in knee articular cartilage from OA rat at different time courses after surgery. *n*=4, **P<0.01. (**g**) Expression and localization (red arrow) of 15-LO-1 were observed by immunohistochemistry staining in OA rat model. *n*=4. (**h**) Quantitative analysis of positive staining. The intensity of the immunostaining of 15-LO-1 was markedly increased in OA rat model. *n*=4, **P<0.01

To further examine the expression of 15-LO-1 in OA, we generated a knee joint OA rat model in which the medial meniscus was resected. MicroCT (*μ*CT) imaging was performed to determine changes in bone architecture in OA rat model (Figure 2a). Hematoxylin–eosin (HE) staining was performed to observe the articular cartilage at the distal femur metaphysic (Figure 2b). Articular cartilage in the sham group of rats possessed regular morphological structure. In contrast, OA rat exhibited evidently the reduction in chondrocytes and articular cartilage thickness with the irregular morphological structure at 12 and 16 weeks after surgery (Figures 2a and b). Furthermore, Toluidine blue staining and Safranin-O/Fast Green staining in knee articular cartilage from OA rat also showed that articular cartilage suffered serious destruction (Figures 2c and d). Subsequently, we observed the expression of 15-LO-1 in knee articular cartilage from OA rat at different time courses after surgery. The results showed that the level of 15-LO-1 was significantly increased in OA rats 12 and 16 weeks after surgery. Furthermore, the increase of 15-LO-1 expression in OA rats was in a time-dependent manner (Figures 2e and f). The intensity of the immunostaining of 15-LO-1 was also markedly enhanced in knee articular cartilage from OA rat (Figures 2g and h).

### The multiple factors associated with the pathogenesis of OA stimulates 15-LO-1 expression in chondrocytes

**Figure 3:**
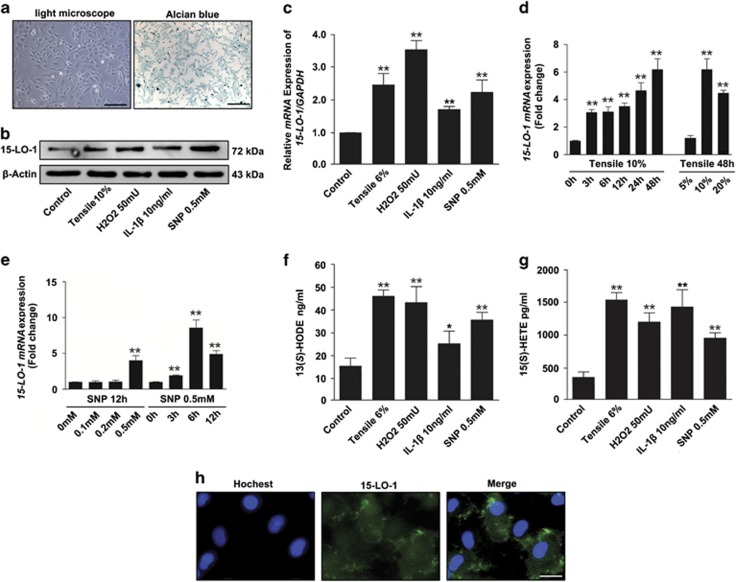
The multiple factors associated with the pathogenesis of OA stimulates 15-LO-1 expression in chondrocytes. (**a**) The chondrocytes isolated from rats were observed by light microscope, and identification of cells was accomplished by Alcian blue staining. Scale bar=50 *μ*m. (**b**) Western blotting analysis of 15-LO-1 expression in chondrocytes treated with 10% tensile, 50 mU H_2_O_2_, 10 ng/ml IL-1*β* and 0.5 mM SNP. *n*=4. (**c**) QRT-PCR analysis of *15-LO-1* expression in chondrocytes treated with 10% tensile, 50 mU H_2_O_2_, 10 ng/ml IL-1*β* and 0.5 mM SNP. *n*=4, **P<0.01. (**d**) QRT-PCR analysis of *15-LO-1 mRNA* expression in chondrocytes treated with tensile at different times and different strengths. *n*=4, **P<0.01. (**e**) QRT-PCR analysis of *15-LO-1 mRNA* expression in chondrocytes treated with SNP at different concentrations and different times. *n*=4, **P<0.01. (**f**) The levels of 13(*S*)-HODE in chondrocytes were measured by enzyme-linked immunosorbent assay (ELISA), which represented activity of 15-LO-1. The multiple factors associated with the pathogenesis of OA could promote the activity of 15-LO-1. *n*=4, *P<0.05, **P<0.01. (**g**) The levels of 15(*S*)-HETE in chondrocytes were measured by ELISA. The multiple factors associated with the pathogenesis of OA could increase the production of 15-(*S*)-HETE. *n*=4, **P<0.01. (**h**) The location of 15-LO-1 in chondrocytes was observed through immunofluorescence. The results showed that 15-LO-1 was mainly distributed in cytoplasm. Scale bar=10 *μ*m

Chondrocytes were isolated from rat knee joints and cells were identified using chondrocyte-specific alcian blue staining (Figure 3a). To further confirm the relationship between 15-LO-1 expression and OA, we observed the effects of multiple factors associated with the pathogenesis of OA, including mechanical loading, ROS, inflammatory factor and nitric oxide (NO), on the expression of 15-LO-1 in chondrocytes. Western blotting and qRT-PCR analysis showed that the expression levels of 15-LO-1 were significantly induced by 10% tensile, 50 mU H_2_O_2_, 10 ng/ml IL-1*β* and 0.5 mM SNP in chondrocytes (Figures 3b and c). Furthermore, we found that mechanical loading stimulated 15-LO-1 expression in time- and strength-dependent manners (Figure 3d). Similarly, upregulation of 15-LO-1 expression by SNP was in concentration- and time-dependent manners (Figure 3e). In addition, 15-LO-1 activity was examined via the measurement of 13(*S*)-HODE production in chondrocytes treated with tensile, H_2_O_2_, IL-1*β* and SNP. We found that the production level of 13(*S*)-HODE were significantly increased in chondrocytes after treatment of tensile, H_2_O_2_, IL-1*β* and SNP, indicating that multiple factors associated with the pathogenesis of OA could promote the activity of 15-LO-1 (Figure 3f). We also measured the level 15(*S*)-HETE, which is a metabolite of arachidonic acid catalyzed by 15-LO-1. The results showed that the levels of 15(*S*)-HETE were unregulated in chondrocytes treated with tensile, H_2_O_2_, IL-1*β* and SNP (Figure 3g). Next immunofluorescence was applied to observe the location of 15-LO-1 in chondrocytes. The results showed that 15-LO-1 was mainly distributed in cytoplasm (Figure 3h).

### 15-LO-1 is involved in the secretion of ECM and cartilage-degrading enzymes

**Figure 4:**
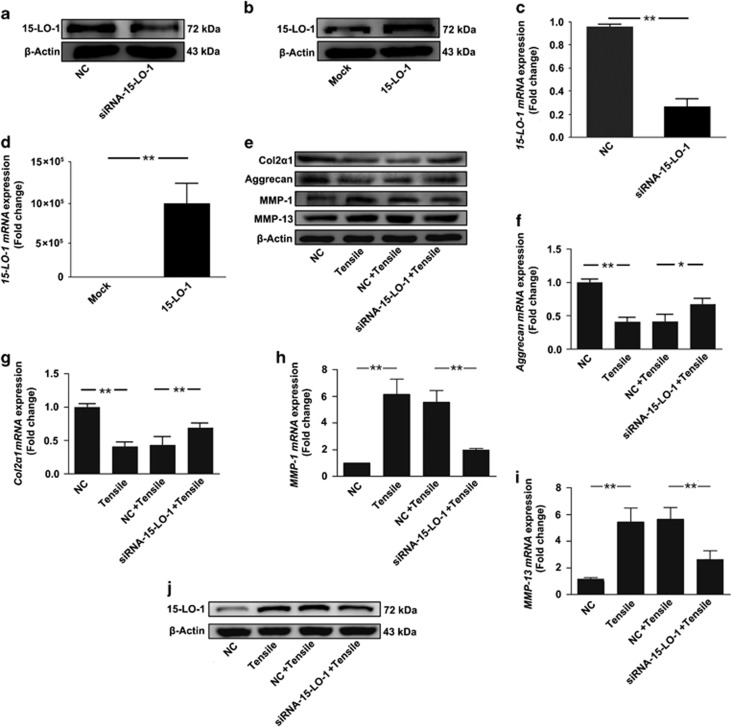
15-LO-1 is involved in the secretion of ECM and cartilage degrading enzymes. (**a** and **b**) Western blotting analysis of 15-LO-1 expression in chondrocytes transfected with siRNA-NC, siRNA-15-LO-1, Mock and plasmid overexpressing 15-LO-1. The results showed that deletion and overexpression of 15-LO-1 were effective. *n*=3. (**c** and **d**) QRT-PCR analysis of *15-LO-1 mRNA* expression in chondrocytes transfected with siRNA-NC, siRNA-15-LO-1, Mock and plasmid overexpressing 15-LO-1. *n*=3, **P<0.01. (**e**) Western blotting analysis of COL2*α*1, aggrecan, MMP1 and MMP13 expression in chondrocytes transfected with siRNA-NC or siRNA-15-LO-1, followed by treating with 10% tensile for 48 h. (**f**–**i**) QRT-PCR analysis of *COL2α1*, *aggrecan*, *MMP-1* and *MMP-13 mRNA* expression in chondrocytes transfected with siRNA-NC or siRNA-15-LO-1, followed by treating with 10% tensile for 48 h. *n*=4, *P<0.05, **P<0.01. (**j**) Western blotting analysis of 15-LO-1 expression in chondrocytes transfected with siRNA-NC or siRNA-15-LO-1, followed by treating with 10% tensile for 48 h

To delineate the role of 15-LO-1 in the secretion of ECM and cartilage-degrading enzymes, the level of 15-LO-1 was decreased with siRNA-15-LO-1 or increased with plasmid overexpressing 15-LO-1. Western blotting and qRT-PCR analysis of 15-LO-1 expression confirmed that the deletion and overexpression of 15-LO-1 were effective (Figures 4a–d). The cartilage ECM consists primarily of aggregating proteoglycans and COL2*α*1. Western blotting and qRT-PCR showed that 10% tensile obviously decreased the expression of aggrecan and COL2*α*1, whereas these were significantly attenuated by silencing of 15-LO-1 (Figures 4e–g). Furthermore, the expression levels of MMP-1 and MMP-13, two critical enzymes for cartilage degrading, were significantly increased in chondrocytes treated with 10% tensile for 48 h. Such changes of MMP-1 and MMP-13 were also alleviated by silencing of 15-LO-1 (Figures 4e, h and i). Successful delivery of siRNA-15-LO-1 and negative control-siRNA (NC) to the chondrocytes under tensile were further verified by comparing the 15-LO-1 levels (Figure 4j). Taken together, these results suggested that 15-LO-1 was involved in the regulation of ECM and cartilage-degrading enzyme secretion by mechanical loading.

### 15-LO-1 inhibits chondrocyte proliferation

**Figure 5:**
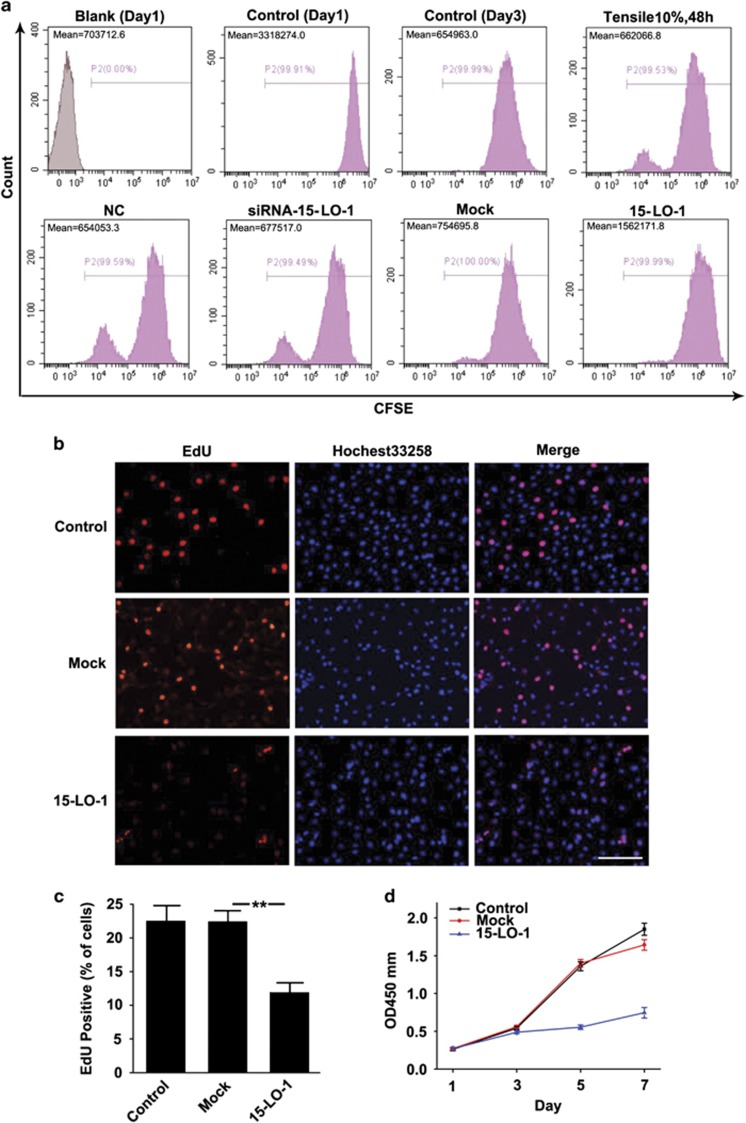
15-LO-1 inhibits chondrocyte proliferation. (**a**) Chondrocytes were transfected with siRNA-NC, siRNA-15-LO-1, Mock and plasmid overexpressing 15-LO-1, stained with CFSE, cultured with 10% tensile for 2 days and analyzed by flow cytometry. CFSE fluorescence intensity is inversely proportional to cells’ proliferation. CFSE fluorescence intensity in different groups is shown in left upper corner. *n*=3. (**b**) Representative photomicrographs of EdU staining. Blue: Hochest labeling of cell nuclei; Red: EdU labeling of nuclei of proliferative cells (× 200). Scale bars=100 *μ*m. (**c**) Quantitative data showing the percentage of EdU-positive cells in different treatment groups (numbers of red *versus* numbers of blue nuclei). *n*=6, **P<0.01. (**d**) Proliferation of cells was measured by CCK-8 in chondrocytes transfected with Mock and plasmid overexpressing 15-LO-1. *n*=6

To examine whether 15-LO-1 was involved in the process of chondrocytes proliferation, chondrocytes were stained with 5,6-carboxyfluorescein diacetate succinimidyl ester (CFSE), which couples irreversibly to cellular proteins. When cells divide, CFSE labeling is distributed equally between daughter cells, which become half as fluorescent as their parents. The peak CFSE fluorescence intensity on flow cytometry was not obviously affected by 10% tensile, suggesting that mechanical loading was not critical for chondrocyte proliferation. Furthermore, only silencing of 15-LO-1 could not significantly promote chondrocyte proliferation, which may be caused by very few expression levels of 15-LO-1 in normal chondrocyte. However, the peak CFSE fluorescence intensity on flow cytometry was shifted in chondrocyte overexpressing 15-LO-1, indicating that cells overexpressing 15-LO-1 had fewer cycles of cell replication as compared with the Mock group (Figure 5a). 5-Ethynil-2′-deoxyuridine (EdU) assay by fluorescence microscope further demonstrated that 15-LO-1 clearly decreased chondrocyte proliferation (Figures 5b and c). Similar results were further confirmed by Cell Counting Kit-8 (CCK-8) assay (Figure 5d). Taken together, these results indicated that 15-LO-1 could inhibit chondrocyte proliferation.

### 15-LO-1 is involved in mechanical loading-induced ROS production

**Figure 6:**
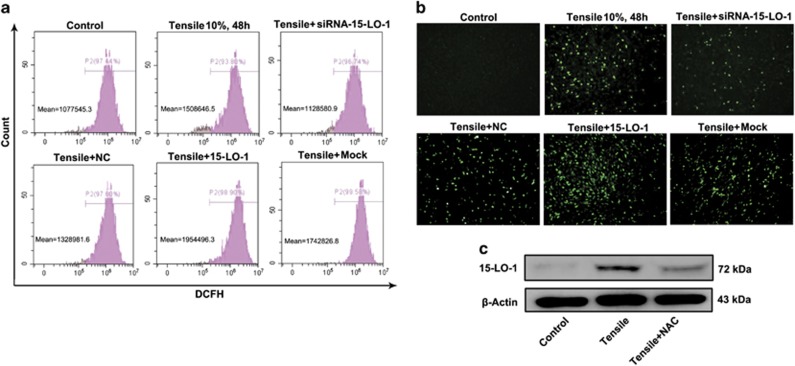
15-LO-1 is involved in mechanical loading-induced ROS production.(**a** and **b**) Chondrocytes were transfected with siRNA-NC, siRNA-15-LO-1, Mock and plasmid overexpressing 15-LO-1, stained with DCF-DA, cultured with 10% tensile for 2 days and analyzed by flow cytometry (**a**) or fluorescence microscope (**b**). *n*=3. (**c**) Western blotting analysis of 15-LO-1 expression in chondrocytes pretreated with NAC, followed by culturing with 10% tensile for 2 days. *n*=3

ROS induced by mechanical loading have an important role in the pathogenesis of OA. To investigate whether 15-LO-1 was involved in mechanical loading-induced ROS production, 2′,7′-dichlorodihydrofluorescein diacetate (DCF-DA) staining was performed to monitor the level of ROS in chondrocyte. The results showed that the peak DCF-DA fluorescence intensity on flow cytometry was increased in chondrocyte treated with 10% tensile for 48 h, suggesting that mechanical loading could promote ROS production. However, induction of ROS by mechanical loading was significantly alleviated in chondrocyte silencing 15-LO-1, whereas overexpression of 15-LO-1 further enhanced tensile-induced ROS production (Figure 6a). In addition, DCF-DA fluorescence intensity was further observed by fluorescence microscope. Consistent with the results from flow cytometry, we found that the enhanced DCF-DA fluorescence intensity was shown in chondrocyte stimulated with 10% tensile for 48 h, which were alleviated by silencing of 15-LO-1 and enhanced by overexpression of 15-LO-1 (Figure 6b). Taken together, these results indicated that 15-LO-1 was involved in mechanical loading-induced ROS production. Next western blotting was used to observe the effect of blocking ROS on tensile-induced 15-LO-1 expression in chondrocyte. We found that NAC, an inhibitor of ROS production, could alleviate tensile-induced 15-LO-1 expression, implicating that the relationship between 15-LO-1 and ROS production may be a positive feedback (Figure 6c).

### 15-LO-1 promotes SNP-induced chondrocyte apoptosis

**Figure 7:**
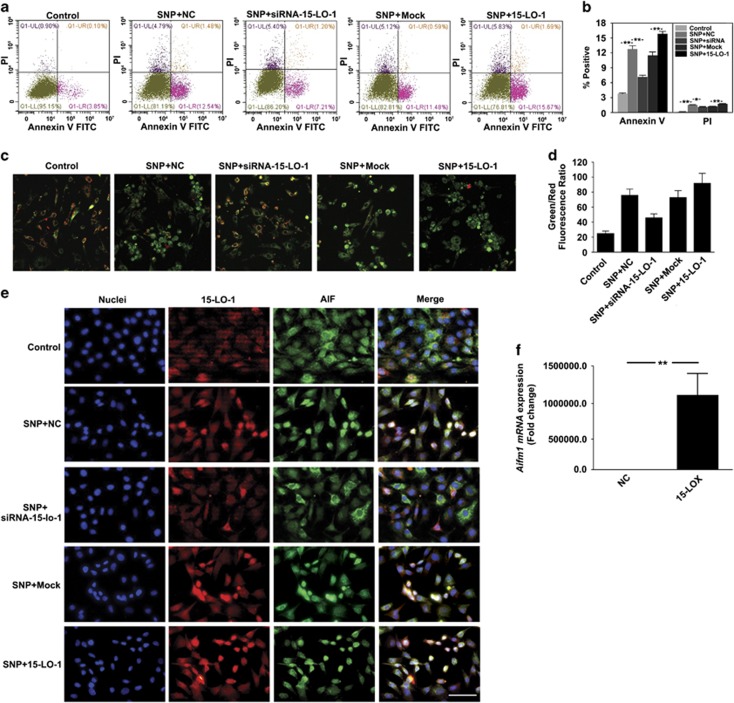
15-LO-1 promotes SNP-induced chondrocyte apoptosis. (**a**) Apoptotic progression was monitored via flow cytometric analysis of phosphatidylserine exposure and plasma membrane integrity. (**b**) The results of flow cytometric analysis are expressed as percentages of positive mean values±S.D., *n*=3, **P<0.01, *P<0.05. (**c**) The cells were stained with JC-1 probe and imaged by a fluorescent microscope. (**d**) The individual red and green average fluorescence intensities are expressed as the ratio of green-to-red fluorescence. The increase of fluorescence ratio, which is represented in the bars, is correlating with an increase in mitochondrial depolarization. (**e**) The expression levels and locations of 15-LO-1 and AIF in chondrocytes, cultured with different conditions, were observed through immunofluorescence. Blue: Hochest labeling of cell nuclei; Red: The expression and location of 15-LO-1. Scale bars=20 *μ*m. Green: The expression and location of AIF. Scale bars=20 *μ*m. (**f**) QRT-PCR analysis of *AIF* expression in chondrocytes transfected with Mock and plasmid overexpressing 15-LO-1. *n*=3, **P<0.01

SNP, as the donor of NO, was used to induce chondrocyte apoptosis. Apoptotic progression was first monitored in chondrocytes via flow cytometric analysis of phosphatidylserine (PS) exposure and plasma membrane integrity. After treatment with 1 mM SNP for 12 h, a population of apoptotic chondrocytes was observed. These cells presented PS externalization as evidenced by a significant increase in annexin-FITC fluorescence, as well as decreased plasma membrane integrity as determined by a modest increase in propidium iodide fluorescence. However, SNP-induced changes were markedly attenuated in chondrocytes silencing 15-LO-1, whereas overexpression of 15-LO-1 further stimulated SNP-induced chondrocyte apoptosis (Figures 7a and b). Subsequently, 5,59,6,69-tetrachloro-1,19,3,39-tetraethylbenzimidazole carbocyanide iodide (JC-1) staining was used to assess the changes in mitochondrial membrane potentials. The quantitative analysis of JC-1-stained cells revealed a significant decrease in the red (high △Ψm) to green (low △Ψm) ratio in SNP-treated cells when compared with control cells. Silencing of 15-LO-1 could obviously increase the red (high △Ψm) to green (low △Ψm) ratio in SNP-treated cells. However, overexpression of 15-LO-1 further promoted the inhibitory effect of SNP on the red (high △Ψm) to green (low △Ψm) ratio in chondrocytes (Figures 7c and d). These results further implicated that 15-LO-1 was involved in SNP-induced chondrocyte apoptosis. Apoptosis-inducing factor (AIF), a bifunctional NADH oxidase, was involved in mitochondrial respiration and caspase-independent apoptosis. QRT-PCR analysis showed that 15-LO-1 could promote *mRNA* expression of *AIF*. Furthermore, the results of immunofluorescence staining showed that the nuclear accumulation of AIF was markedly increased by 15-LO-1. However, silencing of 15-LO-1 was able to obviously attenuate the promontory effect of SNP on the nuclear accumulation of AIF (Figures 7e and f).

### Silencing of 15-LO-1 *in vivo* alleviate destabilized medial meniscus (DMM)-induced OA

**Figure 8:**
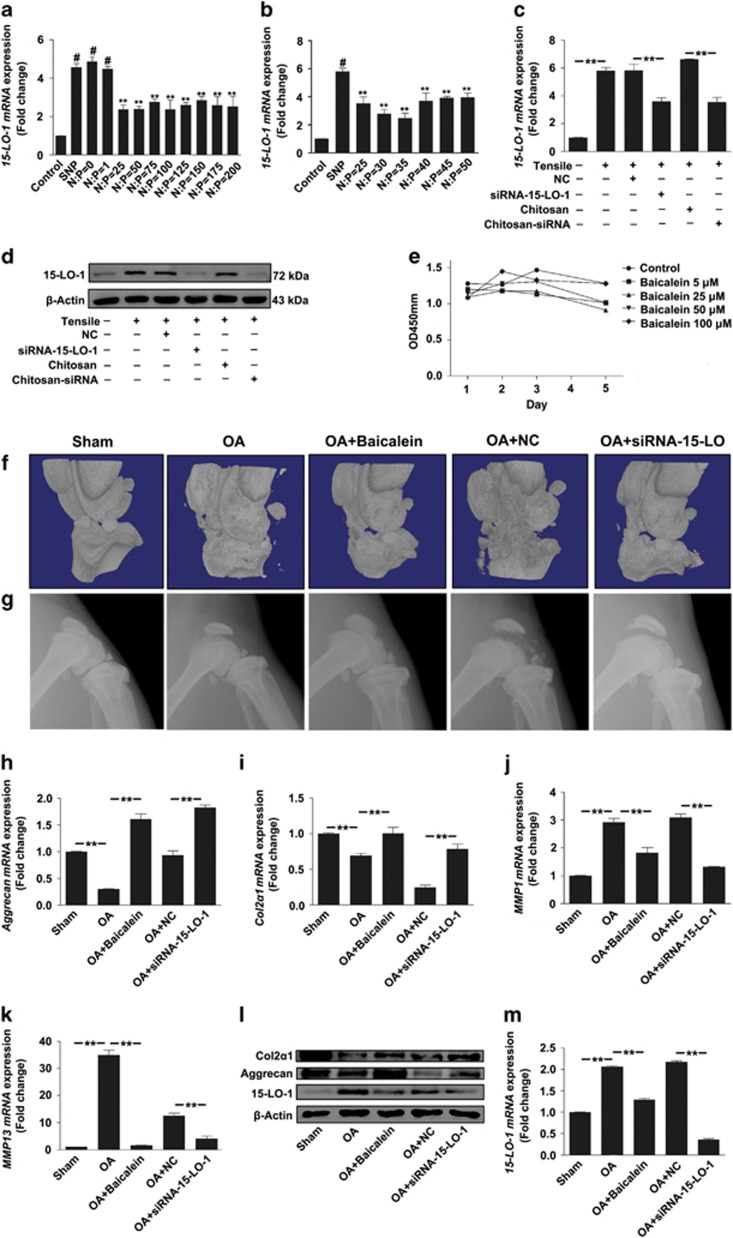
Silencing of 15-LO-1 *in vivo* alleviate DMM-induced OA. (**a** and **b**) QRT-PCR analysis of SNP-induced 15-LO-1 expression in chondrocytes transfected with Chitosan/siRNA-15-LO-1 nanoparticles at different N:P. *n*=3, #*P*<0.01, compared with control; **P<0.01, compared with group SNP. (**c** and **d**) QRT-PCR (**c**) and western blot (**d**) analysis of 15-LO-1 expression in chondrocytes transfected with siRNA-NC, siRNA-15-LO-1, Chitosan and Chitosan/siRNA 15-LO-1, followed by treating with 10% tensile for 48 h. *n*=3, **P<0.01. (**e**) CCK-8 cytotoxicity assay for chondrocytes treated with different concentrations of Baicalein. *n*=6. (**f** and **g**) Micro-CT (**f**) and X-ray (**g**) imaging for morphological structure in the knee of OA rat at 12 weeks after DMM surgery, followed by treatment with intra-articular injection of Baicalein, Chitosan/siRNA-NC and Chitosan/siRNA 15-LO-1, respectively. *n*=4. (**h**–**k**) QRT-PCR analysis of *aggrecan*, *COL2α1*, *MMP1* and *MMP13* expression in the knee articular cartilage from different rat models. *n*=4, **P<0.01. (**l**) Western blotting analysis of *15-LO-1*, *aggrecan* and *COL2α1* expression in the knee articular cartilage from different rat models. *n*=4. (**m**) QRT-PCR analysis of *15-LO-1* expression in the knee articular cartilage from different rat models. *n*=4, **P<0.01

To further investigate the effect of 15-LO-1 on OA *in vivo*, siRNA 15-LO-1 nanoparticles (siRNA-15-LO-1-CH) and baicalein, an inhibitor of 15-LO-1, were intra-articular injected to decrease 15-LO-1 *in vivo*. Various concentrations of chitosan were used to mix with siRNA 15-LO-1 at different N:P ratios (range 0–200) to form chitosan/siRNA nanoparticles. N:P=35 of nanoparticles was finally applied owing to the best inhibitory effect for SNP-induced 15-LO-1 upregulation (Figures 8a and b). In addition, the inhibitory effects of siRNA-15-LO-1-CH on tensile-induced 15-LO-1 expression in chondrocytes were also examined by qRT-PCR and western blotting (Figures 8c and d). Next we performed a CCK-8 assay to examine the effect of baicalein on cells’ viability. The results showed that no significant cytotoxic effects were observed in chondrocytes treated with baicalein, even at concentrations up to 100 *μ*M (Figure 8e). To observe the effect of decreasing 15-LO-1 *in vivo* on OA, *μ*CT imaging was first used to determine changes in bone architecture and bone mineral density in rat model. The reduction of chondrocytes and articular cartilage thickness with the irregular morphological structure were shown in OA rat at 12 weeks after DMM surgery. However, decreasing of 15-LO-1 with siRNA-15-LO-1-CH or baicalein could obviously attenuate DMM-induced OA (Figure 8f). X-ray examination showed better integrity of articular surface and less bone destruction and osteophyte formation on either siRNA-15-LO-1-CH or baicalein treatment compared with the control group, respectively (Figure 8g). QRT-PCR analysis showed the reduction of *aggrecan* and *COL2α1 mRNA* expression in the knee articular cartilage from OA rats was obviously alleviated by inhibition of 15-LO-1 *in vivo* (Figures 8h and i). The *mRNA* expression levels of *MMP-1* and *MMP-13* were lower in the knee articular cartilage from OA rats compared with OA rats treated with siRNA-15-LO-1-CH or baicalein, implicating that blocking of 15-LO-1 *in vivo* could alleviate OA (Figures 8j and k). Similar results were further demonstrated by western blotting (Figure 8l). 15-LO-1 mRNA expression levels were observed in articular cartilage from different group of rats. The results showed that siRNA-15-LO-1-CH or baicalein injection could obviously decrease 15-LO-1 *in vivo* (Figure 8m), suggesting that the results from rat models were reliable.

## Discussion

Upregulation of 15-LO-1, leading to increasing of 15-HETE production, has been confirmed to exacerbate inflammation and oxidative stress in many diseases.^16, 17, 18^ However, it was unclear whether 15-LO-1 was involved in the pathogenesis of OA. Results of the present studies for the first time demonstrated that the level of 15-LO-1 expression was significantly increased in OA. Further studies showed that 15-LO-1 was involved in the regulation of chondrocyte functions by multiple factors associated with OA, including ECM secretion, cartilage-degrading enzyme secretion, the ROS production, chondrocytes proliferation and apoptosis. In addition, reduction of 15-LO-1 *in vivo* could markedly alleviate OA. Taken together, these results indicated that 15-LO-1, a crucial regulator for chondrocyte function, might be used as therapeutic target for OA.

Abnormal joint loading caused by instability or injury of the joint has emerged as a critical risk factor for the onset and progression of OA.^19^ Previous studies showed that impact loads could lead to significant damage to the articular cartilage, such as splitting of the ECM, increased cellular activity, increased tissue hydration and remodeling of the subchondral bone,^20^ which are generally consistent with the early stage of OA. Thus we first explored whether abnormal of joint loading could affect the 15-LO-1 expression. We found that the expression of 15-LO-1 was significantly increased in the articular cartilage from OA patients, especially in the weight-bearing area of articular cartilage, indicating that abnormal joint loading could promote 15-LO-1 expression. To further examine this result, we observed the effect of mechanical loading on 15-LO-1 expression in chondrocyte *in vitro.* The results showed that mechanical loading stimulated 15-LO-1 expression in time- and strength-dependent manners. To our knowledge, the present study is the first effort to observe the effect of biomechanical loading on 15-LO-1 expression in OA. However, the exact mechanisms by which biomechanical loading regulate the 15-LO-1 expression need to be further explored in the future study.

Indeed, the expression of 15-LO-1 in the articular cartilage from OA has been investigated in the previous study.^13^ Chabane *et al.*^13^ found that 15-LO-1 was expressed in the articular cartilage from OA patients, but they did not measure the expression of 15-LO-1 in the articular cartilage from normal human. Furthermore, they confirmed that 15- HETE, the 15-LO-1 metabolite, suppressed IL-1*β*-induced MMP-1 and MMP-13 expression in a PPAR*γ*-dependent pathway. Thus they concluded that 15-LO-1 may have chondroprotective properties by reducing MMP-1 and MMP-13 expression. In contrast to the study by Chabane *et al.*,^13^ we measured the expression of 15-LO-1 in articular cartilage not only from OA patients but also from normal humans. We found that the expression of 15-LO-1 was significantly increased in OA patients compared with normal humans. In addition, we demonstrated that 15-LO-1 could obviously promote biomechanical loading-induced-MMP-1 and MMP-13 expression and biomechanical loading-induced ROS production. In contrast, 15-LO-1 could significantly alleviate NO-induced chondrocyte apoptosis and chondrocyte proliferation. Taken together, our studies suggested that 15-LO-1 may be a critical factor for the pathogenesis of OA. Our conclusions were different from the conclusion reached by Chabane *et al.*^13^ It was possible that we directly observed the effects of silencing or overexpressing 15-LO-1 on chondrocyte function induced by various factors associated with OA. However, Chabane *et al.*^13^ investigated the effects of 15-LO-1 metabolites, 13-HODE and 15-HETE, on proinflammatory cytokines-induced MMP-1 and MMP-13 expression. In addition, owing to the diversity and complexity of substrates catalized by 15-LO-1, the roles and exact mechanisms of 15-LO-1 metabolites in OA need to be further explored in future studies.

Previous studies showed that excessive articular cartilage loading triggered release of ROS from mitochondria and that these ROS cause chondrocyte death and matrix degradation.^21, 22^ Therefore, preventing ROS release or inhibiting their effects could preserve chondrocytes and their matrix. In the present study, we found that biomechanical loading significantly induced ROS production, which were consistent with the results from previous studies. There have been a few studies confirming that 15-LO-1 metabolites of arachidonic acid, particularly 15(*S*)-HETE, could stimulate the ROS production, leading to many cells’ damage.^14, 23^ Similarly, we found that silencing 15-LO-1 could obviously alleviate biomechanical loading-induced ROS production, indicating that the induction of ROS release by biomechanical loading was mediated by 15-LO-1. Furthermore, our results showed that NAC, an inhibitor of ROS, attenuated tensile-induced 15-LO-1 expression, implicating that the relationship between 15-LO-1 and ROS production may be a positive feedback.

Increasing amounts of evidence has shown that chondrocyte apoptosis have great roles in cartilage development, aging and disease.^24^ NO has emerged as a key factor for mediation of chondrocyte apoptosis in OA.^25^ It has been suggested that either endogenous or exogenous NO can induce apoptosis in chondrocytes via a mitochondria-dependent mechanism. SNP is generally used as NO donor to study the role of NO in chondrocytes apoptosis.^26^ In our study, we found that silencing 15-LO-1 significantly alleviated SNP-induced chondrocyte apoptosis, suggesting that 15-LO-1 have an important role in chondrocyte apoptosis. The effects of 15-LO-1 on cells apoptosis have been observed in various cells, such as prostate cancer cells, breast cancer cell and pulmonary artery smooth muscle cells. Our study investigated the role of 15-LO-1 in chondrocyte apoptosis. The exact mechanisms by which 15-LO-1 inhibited chondrocyte apoptosis need to be further investigated.

In addition to confirming the roles of 15-LO-1 in chondrocyte function *in vitro*, we also observed the effects of 15-LO-1 decreasing on OA *in vivo*. Both chemistry and biology inhibitors were dividedly used to reduce the expression of 15-LO-1 *in vivo.* Baicalein, as a flavonoid isolated from *Scutellaria baicalensis Georgi*, has been demonstrated to possesses antioxidant and anti-inflammatory properties.^27, 28^ In other way, chitosan has been widely used in drug delivery systems, due to its biocompatible and nontoxic.^29^ The present results showed that the transfection efficiency of chitosan was significantly higher compared to lipofectamine 3000. Consequently, we chose chitosan as a siRNA-15-LO-1 carrier *in vivo*. Reduction of 15-LO-1 *in vivo* by these two manners provide strong evidence that inhibiting 15-LO-1 *in vivo* could obviously alleviate OA, suggesting that 15-LO-1 may be a therapeutic target for OA.

In conclusion, our study provided new evidence that 15-LO-1 is a crucial factor for the initiation of OA. In the present study, we found that silencing 15-LO-1 could decrease the secretion of ECM and cartilage-degrading enzymes. Furthermore, 15-LO-1 was involved in the process of chondrocyte apoptosis and proliferation. Moreover, inhibition of 15-LO-1 *in vivo* could significantly alleviate DMM-induced OA. This study may provide new insights into the development of pharmacological and physical therapies that can modify the course of OA.

## Materials and methods

### Human knee cartilage procurement

Knee cartilage samples were obtained from trauma donor who needed lower limb amputation and OA patients who underwent total knee arthroplasty. Relative non-weight bearing/weight-bearing area cartilage procurement was performed as previously reported.^30^ Pieces of smooth cartilage resected from the area with surface integrity without any irregularity (no staining with India ink, smooth cartilage) were regarded as relative non-weight bearing area, while pieces of damaged cartilage obtained from the area with gross erosions and stained positive with India ink were regarded as weight-bearing area. Ethical approval was obtained from the Shanghai Ruijin Hospital review board for human knee cartilage samples. All patients gave informed consent.

### Animal experiments

Adult male Sprague-Dawley rats were used to induce OA model by DMM surgery (5 groups and in each group *n*=6; 8-week old; mean body weight=220 g). Briefly, after anesthetization, a medial capsular incision was made to expose the right knee joint. Then the medial meniscotibial ligament was transected, and the medial meniscus could be displaced medially. Finally, the medial capsular incision was sutured, and the skin was closed. A sham operation was performed in another group by only opening the joint cavity. After wound healing, chitosan (Sigma-Aldrich, St. Louis, MO, USA)/siRNA-15-LO-1-CH were formulated as previously described with the final N:P=35.0.^31^ Then intra-articular injection was performed with 200 *μ*l nanoparticles (0.5 nmol siRNA) twice a week. In addition, 1 mg baicalein (Cayman Chemicals, Ann Arbor, MI, USA) per knee, an inhibitor of 15-LO-1, was intra-articular injected, whereas saline injection was used as control. All animal experiments were performed according to the protocol approved by the Shanghai Jiao Tong University (SJTU) Animal Care and Use Committee.

### Radiological evaluation

Knees of OA rats were received to radiographical evaluation at every time point. MX-20 Cabinet X-ray System (Faxitron, Tucson, AZ, USA) was used to acquire digital plain radiographs of the morphology of rat knees before killing by excessive anesthesia. Then intact knee joints were dissected out of soft tissue and fixed in 70% ethanol for 24 h. Samples were scanned using SkyScan1172 high-resolution micro-CT (Bruker, Kontich, Belgium) as previously described,^32^ with some modifications that set the parameters as follows: 100 kVp, 100 *μ*A, and 10.0 *μ*m per pixel. The data were reconstructed and three-dimensional modeled for further analysis.

### Histological analysis

Samples were fixed in 4% paraformaldehyde, followed by decalcification in EDTA-buffered saline solution (pH 7.4, 0.25 M). Tissue sections were then cut longitudinally to obtain 10 *μ*m sections. Histological changes and proteoglycan/collagen content were observed by HE, Safranin-O/Fast Green and Toluidine blue staining.

### Immunohistochemistry

The paraffin-embedded tissues were used for the immunohistochemical analysis of 15-LO-1 expression in the articular cartilage from patients and rat. Full-thickness specimens were processed for immunohistochemical analysis, as previously described.^4^ Briefly, after the slides were incubated with a blocking serum (Vectastain ABC Kit; Vector Laboratories, Inc., Burlingame, CA, USA) for 60 min, they were blotted and then overlaid with the primary antibody against 15-LO-1 for 2 h at room temperature. Subsequently, biotinylated secondary antibodies were added into the sections, followed by a peroxidase-labeled streptavidin–biotin staining technique (DAB Kit, Invitrogen, Paisley, UK).

### Chondrocyte culture and tensile strain loading

Chondrocytes were isolated from articular cartilage in the knee joints of rats as previously reported.^4^ Articular cartilage tissues were cut into small pieces (<1 mm^3^) and digested with 0.25% trypsin for 30 min, followed by digestion with 0.2% type II collagenase for 4 h. The released cells were cultured in DMEM/F12 media supplemented with 10% fetal bovine serum (FBS) and antibiotics. Once reached to 80% confluence, the cells were subjected to cyclic tensile strain with a 0.5 Hz sinusoidal curve at 5–20% elongation for 12–48 h using an Flexcell1 FX-5000 Tension System as described in the manufacturer’s manual (Flexcell International Corporation, Burlington, NC, USA). Only cells with less than two passages were used in order to preserve chondrocyte phenotype.

### Cell transfection

The overexpression plasmid vector for rat 15-LO-1 gene was created from CMV-MCS-EGFP-SV40-Neomycin constructed by Genechem (Shanghai, China). The candidate siRNA duplexes against rat15-LO-1 were synthesized from GenePharma (Shanghai, China) with the sequences: sense 5′-GGAUGUGUCAGGAUACCU UTT-3′, and antisense 5′-AAGGUAUCCUGACACAUCCTT-3′. Chondrocytes were inoculated into six-well tissue culture plates at a density of 2 × 10^5^ cells in 2 ml of standard growth media per well and incubated for 24 h prior to transfection. Once chondrocytes reached approximately 80% confluence, cells were transfected with 100 nM siRNA or 4 *μ*g plasmid DNA using Lipofectamine 3000 reagent (Invitrogen) according to the manufacturer’s protocols. Cells infected with negative control siRNA or empty vector (Mock) worked as control, separately.

### Western blotting analysis

Western blotting analysis was accomplished to examine the expression of 15-LO-1 according to a previous report.^33^ Briefly, 15 *μ*g of plasma proteins were extracted from chondrocytes using 10% SDS-PAGE. Proteins were electroblotted onto a polyvinylidene difluoride membrane (0.45 mm; Millipore, Bedford, MA, USA), followed by blocking using 5% non-fat dry milk in Tris-buffered saline with Tween 20 for 1 h. The membranes were then incubated at 4 °C overnight with anti-15-LO-1 antibodies (ratio of antibodies to milk of 1 : 500), anti-COL2*α*1 antibodies (1 : 400), anti-Aggrecan antibodies (1 : 1000), anti-MMP-1 antibodies (1 : 500) and anti-MMP-13 antibodies (1 : 500) (all from Santa Cruz Biotechnology, Santa Cruz, CA, USA), followed by incubation with HRP-conjugated secondary antibodies (1 : 4000) (Santa Cruz) at room temperature for 1 h. Enhanced chemiluminescence assay (Thermo Scientific, Pierce, Rockford, IL, USA) was used to visualize antigen–antibody complexes.

### Quantification of mRNA and qRT-PCR

Total RNA from chondrocytes was extracted using TRIzol reagent (Invitrogen) as previously reported.^4^ cDNA was synthesized using 1 *μ*g of RNA and a RevertAid First Strand cDNA Synthesis Kit (TaKaRa, Dalian, China). QRT-PCR was performed to amplify the cDNA using the SYBR Premix Ex Tag Kit (TaKaRa) and an ABI 7500 Sequencing Detection System (Applied Biosystems, Foster City, CA, USA). The primer sequences used in this study are described in Table 1.

### Measurement of 15-LO-1 activity and metabolites by enzyme-linked immunosorbent assay

According to previous reports,^10, 34^ the 15-LO-1 activity could be evaluated through measurement of 13(*S*)-HODE production, which is the major 15-LO-1 product derived from linoleic acid oxidation. 15-LO-1 activity assay was accomplished as previous studies with slight modification.^34^ Briefly, the chondrocytes after treatment of tensile, H_2_O_2_, IL-1*β* and SNP were lysed by using the 13(*S*)-HODE EIA Kit (Enzo Life Science, Farmingdale, NY, USA), followed by incubating with 18 *μ*M concentration of linoleic acid in ethanol at 37 °C for 45 min. The reaction was terminated by 0.2 M HCl. The production level of 13(*S*)-HODE in cells was measured by using the 13(*S*)-HODE EIA Kit. Similarly, the cell lysates were incubated with arachidonic acid, and then the level of 15-HETE was measured by 15(*S*)-HETE EIA Kit (Cayman Chemicals).

### CCK-8 assay

The effects of 15-LO-1 on chondrocyte viability were determined using a CCK-8 assay (Dojindo Laboratories, Kumamoto, Japan) according to the manufacturer’s instructions. Briefly, chondrocytes were inoculated at a density of 2 × 10^3^ per well into 96-well plates and cultured at 37 °C in 5% CO_2_. 10 *μ*L WST-8 was added into each well at 37 °C in 5% CO_2_ for 1 h. The absorbance of each sample was measured at a wavelength of 450 nm.

### EdU incorporation assay

Chondrocytes were inoculated at a density of 2 × 10^5^ per well into 24-well plates and cultured at 37 °C in 5% CO_2_. 50 *μ*M of EdU (Sigma-Aldrich) was then added to each well for 2 h. Next, cells were fixed with 4% formaldehyde for 15 min, followed by permeabilization with 0.5% Triton X-100 for 20 min at room temperature. After washing the cells three times with PBS, 100 *μ*l of 1X Apollo reaction cocktail was added to each well for 30 min at room temperature. Subsequently, the cells were stained with Hoechst 33258. The EdU incorporation rate was expressed as the ratio of EdU-positive cells (red cells) to total Hoechst 33342-positive cells (blue cells).

### CFSE assay

Chondrocytes were labeled with 2.5 *μ*M CFSE (Invitrogen) at 37 °C for 10 min, followed by washing twice with ice-cold PBS. CFSE-labeled chondrocytes were then inoculated into six-well plates and cultured in DMEM/F-12 media containing 10% FBS. Cells were harvested on day 5 and fluorescence intensity was measured by flow cytometry. The results were analyzed with the Cell Quest software (BD Bioscience Pharmingen, San Jose, CA, USA).

### Detection of the intracellular ROS levels

The fluorescent dye DCF-DA (Invitrogen) was used to detect the intracellular ROS levels in rat chondrocytes. Chondrocytes were centrifuged and resuspended in serum-free buffer containing 10 *μ*M DCF-DA for 30 min at 37 °C. No-dye control was used to eliminate background fluorescence. The fluorescence intensity was measured by fluorescence microscope or flow cytometry.

### Flow cytometric staining

Chondrocyte apoptosis was measured by flow cytometry as previously reported.^35^ Briefly, cells were washed in 4 °C PBS, centrifuged and resuspended in 0.5 ml of hypotonic fluorochrome solution containing 50 *μ*g/mL propidium iodide (PI), 0.1% sodium citrate and 0.1% Triton X-100 (Sigma-Aldrich) to quantitate the cellular DNA content under the permeabilized condition. The presence of PS due to flipping of the plasma membrane, a concomitant feature during apoptosis, was evaluated by annexin V-FITC staining. Cells were washed with PBS and incubated in a solution of 0.5 *μ*g/ml FITC-labeled annexin V. At the same time, cells were stained by the PI exclusion method for the detection of dead cells. Cells were then analyzed by flow cytometry.

### Immunofluorescence staining

Chondrocytes were inoculated into culture slide (Corning, Corning, NY, USA) to facilitate microscopic observation. After 4% paraformaldehyde fixation, 0.5% Triton X-100 permeabilization and serum blocking as previously described,^36^ cells were incubated in the mixture of rabbit polyclonal anti-15-LO-1 antibodies (1 : 500) and mouse monoclonal anti-AIF antibodies (1 : 500) (Santa Cruz) at 4 °C overnight and then incubated with the mixture of two secondary antibodies at room temperature for 1 h, which are mouse IgG (H+L) secondary antibodies (Alexa Fluor 488, 1 : 1000) and rabbit IgG (H+L) secondary antibodies (Alexa Fluor 555, 1 : 1000) (Invitrogen).

### Determination of mitochondrial membrane potential (△Ψm)

The JC-1 (Invitrogen) was used to measure the △Ψm of chondrocytes. In the high △Ψm, JC-1 gathered in the mitochondrial matrix to form polymers (J-aggregates, red fluorescence), while it existed as a monomer at low values of △Ψm (green fluorescence). Consequently, mitochondrial depolarization occurring in apoptosis could be indicated by a decrease in the red/green fluorescence intensity ratio. Briefly, chondrocytes were inoculated into culture slides and transfected with Alox-15 siRNA or plasmid DNA before inducing apoptosis by 1 mM SNP for 12 h. Then cells were incubated with 2 *μ*M JC-1 at 37 °C in 5% CO_2_ for 15 min, washed in PBS and observed by fluorescence microscope with 488 and 633 nm excitation.

### Statistical analysis

Data were collected from three or more independent experiments and expressed as mean±S.D. A two-sided Student’s *t*-test was used to analyze the difference between groups. One-way analysis of variance was performed to show the difference between groups. *P*<0.05 was considered significantly different.

## Publisher’s Note

Springer Nature remains neutral with regard to jurisdictional claims in published maps and institutional affiliations.

## Figures and Tables

**Table 1:** Primer sequences used for qRT-PCR in this study

**Organism**	**Targets**	**Forward primer/reverse primer**	**Genbank accession no.**
Human	*β-Actin*	Forward 5′-ATCAGTGGGTGCCTTACCAA-3′ Reverse 5′-GCCAAATGTTTTACTGGGACA-3′	NM_001101.3
Human	*15-LO-1*	Forward 5′-TTCTGTCCCCCTGATGACTT-3′ Reverse 5′-ACGATTCCTTCCACATACCG-3′	NM_001140.3
Rat	*β-Actin*	Forward 5′-TGTCACCAACTGGGACGATA-3′ Reverse 5′-GGGGTGTTGAAGGTCTCAAA-3′	NM_031144.3
Rat	*15-LO-1*	Forward 5′-CCACCTGGATCTTCTCAAGC-3′ Reverse 5′-GCAGGGCGTCTTTAGCATAG-3′	NM_031010.2
Rat	*Acan*	Forward 5′-GATCTCAGTGGGCAACCTTC-3′ Reverse 5′-TCCACAAACGTAATGCCAGA-3′	NM_022190.1
Rat	*Col2a1*	Forward 5′-CTCAAGTCGCTGAACAACCA-3′ Reverse 5′-GTCTCCGCTCTTCCACTCTG-3′	NM_012929.1
Rat	*MMP-1*	Forward 5′-CACTCCCTTGGACTCACTCA-3′ Reverse 5′-CCCATATAAAGCCTGGATGC-3′	NM_001134530.1
Rat	*MMP-13*	Forward 5′-TGGCGACAAAGTAGATGCTG-3′ Reverse 5′-TGGCATGACTCTCACAATGC-3′	NM_133530.1
